# Alaska Native Elders’ perspectives on dietary patterns in rural, remote communities

**DOI:** 10.1186/s12889-021-11598-8

**Published:** 2021-09-09

**Authors:** Amanda K. Walch, Kathryn A. Ohle, Kathryn R. Koller, Lucinda Alexie, Flora Sapp, Timothy K. Thomas, Andrea Bersamin

**Affiliations:** 1 Dietetics & Nutrition Department, University of Alaska Anchorage, 146 Professional Studies Building, 3211 Providence Drive, Anchorage, AK 99508 USA; 2 Early Childhood Education, Grand Valley State University, 441C Richard M. DeVos Center, 401 Fulton St. W, Grand Rapids, MI 49504-6431 USA; 3 Clinical & Research Services, Alaska Native Tribal Health Consortium, 3900 Ambassador Dr., Ste. 201, Anchorage, AK 99508 USA; 4 Division of YKHC Medical Director, Yukon Kuskokwim Health Corporation, Post Office Box 528, Bethel, AK USA; 5 Center for Alaska Native Health Research, Institute of Arctic Biology, University of Alaska Fairbanks, PO BOX 757000, Fairbanks, AK 99775 USA

**Keywords:** Alaska Native, Traditional foods, Elders, Diet, Childhood obesity

## Abstract

**Background:**

Given the increasing rates of childhood obesity in Alaska Native children and the understanding that the most effective interventions are informed by and reflect the cultural knowledge of the community in which they are implemented, this project sought to gather the wisdom of local Yup’ik and Cup’ik Elders in the Yukon-Kuskokwim region of Alaska around how to maintain a healthy diet and active lifestyle.

**Methods:**

Perspectives were sought through the use of semi-structured focus groups, which were completed in person in twelve communities. All conversations were recorded, translated, transcribed, and analyzed using a qualitative approach, where key themes were identified.

**Results:**

Elders provided a clear and consistent recollection of what their life looked like when they were young and expressed their perspectives related to maintaining a healthy and traditional lifestyle. The key themes the Elders discussed included an emphasis on the nutritional and cultural benefits of traditional foods; concerns around changing dietary patterns such as the consumption of processed foods and sugar sweetened beverages; and concerns on the time and use of screens. Elders also expressed a desire to help younger generations learn traditional subsistence practices.

**Conclusions:**

The risk of obesity in Alaska Native children is high and intervention efforts should be grounded in local knowledge and values. The perspectives from Yup’ik and Cup’ik Elders in the Yukon-Kuskokwim Delta area of Alaska provide a better understanding on local views of how to maintain a healthy diet, physical activities, and traditional values.

## Background

Childhood obesity has increased three-fold across all races and ethnicities during the past 30 years [1, 2]. Obesity is most prevalent among Hispanic and Alaska Native/American Indian (AN/AI) peoples, with increases in early childhood obesity particularly evident among AN/AI children [3, 4]. Unpublished data collected for reporting purposes by one organization managing Head Start programs in Alaska’s rural Yukon-Kuskokwim (Y-K) region, showed 68% of 3- to 5-year old AN children met criteria for being overweight according to the Centers of Disease Control and Prevention’s growth charts, while 43% met criteria for being obese. Substantial evidence suggests that obesity in early childhood, from birth to age 5 years, is a strong predictor of adolescent and adult obesity [2, 5–8]. In two cohort studies conducted within Alaska’s Y-K region during 2004–2010, approximately 40% of Alaska Native (AN) adults were obese [9]. These data collectively emphasize the importance of establishing healthy eating and physical activity practices at an early age as the primary prevention method for obesity in adulthood.

Research suggests that obesity prevention efforts are most effective in Indigenous communities when they are aligned with the cultural values, norms, and strengths of the local nutrition environment [10–12]. Because local understandings of food and health in Indigenous communities arise from integrated, holistic, place-based worldviews that are connected to local value systems, it is important for prevention efforts to reflect these traditional values [13, 14]. Specifically, nutrition interventions in Indigenous communities need to address the way local perceptions and understandings of how food affects “the physical, psychological, social, and spiritual dimensions of all age and gender groups in community life” [15]. In Alaska, rural remote communities are isolated, located off the road system, and predominantly populated by AN people indigenous to the region. To date, the only known successful, small-scale, nutrition intervention conducted and published in the Y-K region, home to AN Yup’ik and Cup’ik people, intentionally integrated cultural values, norms, and strengths of the local nutrition environment [15].

AN Yup’ik communities have great respect for their Elders as knowledge bearers [16]. Elders pass traditional values, norms, beliefs, and practices down through the generations via storytelling and practical application and are therefore well positioned to pass on knowledge about the local nutrition environment in rural Alaska [17, 18]. However, in just the past few decades, massive social, political, and economic movements have disrupted the intergenerational transmission of traditional ways of life that have sustained Alaska’s isolated communities for centuries. Exposure to external cultures through colonization, westernization, mechanization, and globalization has contributed to an immense loss of Indigenous knowledge [17–19]. In addition, many of these same factors have caused a shift in the traditional food environment and contributed to a nutrition transition [20]. While many Elders living in these communities still possess traditional wisdom about healthy diet and lifestyle practices, this wisdom may not be known or followed by young families [21, 22].

With the elevated risk of obesity in AN children, prevention efforts are underway to help curtail the trend toward obesity, including two studies that are led by the authors of this paper and funded by the National Institutes of Health (R01NR015417) and the U.S. Department of Agriculture (2018–69,001-27,544). Elders can aid in the development of these efforts by sharing their personal journey through the traditional norms of the past to the present norms today. To enhance the local and regional relevance to design, implement, and evaluate an obesity prevention effort, the objective of this study was to listen to Yup’ik and Cup’ik Elders to better understand their views on maintaining a healthy diet, physical activities, and traditional values to inform obesity prevention efforts. These objectives fit into the goals of the broader intervention plan to 1) increase the consumption of nutrient dense traditional foods, and fruits and vegetables from the tundra and the store, while decreasing high-calorie low-nutrient prepared foods; 2) decrease the consumption of sugar-sweetened beverages, while increasing water intake; and 3) decrease screen time while increasing physical activity.

## Methods

The study was reviewed and approved by the Alaska Area Institutional Review Board, and Tribal approval bodies at the Alaska Native Tribal Health Consortium and the Yukon-Kuskokwim Health Corporation. Investigators carried out the exploratory qualitative study in May through December 2018, in preparation for a large scale, randomized community intervention designed to promote healthy eating and physical activity.

### Setting

The research team conducted 12 Elder focus groups in 12 rural, remote Alaska Native communities in the Yukon-Kuskokwim Delta region in southwest Alaska. These communities, with populations that range between 300 and 1200 people, are located off the road system, which means that they are accessible only by small aircraft year-round and by boat in the summer and snow machine in the winter. Permanent residents in the communities are primarily Yup’ik/Cup’ik ethnicity, whose lifestyles are based on mixed economies of subsistence practices (e.g., hunting, fishing, and gathering) blended with some cash income, which can be supplemented by federal assistance programs [23, 24]. This region has one of the lowest population densities in the US and has limited infrastructure [25].

### Participants

Respecting requests made by community members and Tribal Councils, two community liaisons were hired to invite Yup’ik/Cup’ik Elders to participate in a focus group. An Elder is considered a community member who could provide cultural insight on the traditional lifestyle. Elders’ command of English varies by individual. In general, most children and young adults speak fluent English, attending schools where instruction is in English. While Elders may speak English, it is a second language for them and many are more comfortable conversing in their first language. Thus, two AN Yup’ik/Cup’ik-speaking interpreters were available to travel with the research team, assisting with translation and interpretation during the focus group discussions. Focus groups were facilitated by non-Native researchers with focus group experience, well-versed in AN cultural values, and deep respect for Elders. Facilitators relied on cues from interpreters signaling an appropriate time to move to the next discussion point. The team did not collect any identifying information from participants. All participants received a gift card at the end of the session.

### Data collection

Focus group questions sought perspectives on the importance of traditional foods and physical activities, as well as barriers to traditional ways. Facilitators asked Elders to provide their opinions to the following questions: “In what ways are traditional foods important?”, “What is the best way to teach about traditional foods?” “Tell me about mealtimes as a child”, “What kinds of activities did children do before television?” and “What concerns do you have about how children are raised today in terms of the foods they eat and the activities they do?” A Yup’ik translator was present at some of the focus groups, when available. All focus group discussions were audio-recorded, translated to English when necessary by the Yup’ik/Cup’ik-speaking interpreters, and transcribed.

### Data analysis

Two researchers listened to each recording and read each transcript. They independently created a summary and a subsequent reflective memo that captured the most salient themes that emerged from the data, using open coding [26]. The memos were then compared and contrasted until consensus was reached by the researchers on the most salient themes. After reaching consensus, the researchers created summative documents by question and themes for each community. Finally, the researchers applied selective coding to the transcripts using the major themes that emerged during the first round of open coding [26]. The themes included the nutritional and cultural benefits of traditional foods, concerns about changing dietary patterns, and suggestions to maintain subsistence practices.

## Results

Focus group sizes varied from three to twelve participants in each focus group. In total, 66 Elders (36 women, 30 men) participated across the 12 sites. Discussions ranged between 90 and 120 min in length. Elders attending the focus groups provided a clear and consistent recollection of what their life looked like when they were young. They also discussed the nutritional and cultural benefits of traditional foods, concerns around changing dietary patterns, and suggestions for maintaining subsistence practices.

### Nutritional benefits of traditional foods

Elders believe strongly in the importance of eating traditional foods, such as seal oil, a variety of fish, berries, moose, and tundra greens. They also cited the importance of consuming fermented foods. These foods were consumed when they were young and the Elders believe they should continue to be consumed today because of their health benefits since, “We know how to take care of ourselves already nutritiously. We’ve done it for many, many, many, many, many, many years” (Alaska Native Village (ANV) 2). To Elders, traditional foods are synonymous with healthy foods and what people should be eating. As one commented, “And the doctors will say to the person who has illness, ‘Eat right food’. Native people, we know what to eat, right food, our own food” (ANV9). This understanding - that traditional foods are the “right food” or the “healthful foods” - was passed down by their ancestors.

*Our ancestors used to say that our Native foods are really good to eat. They are the healthful foods. And also the berries. And also the plants that grow around this land. They’re really good. And some are medicines, like the plants. And then as I was growing up I came upon two people who became sick and they said that they blamed the preservatives that are in canned food* (ANV9).

Elders also recognize the short and long-term benefits of traditional foods. For example, one Elder, recognizing the immediate impact of traditional foods, shared that “He [dad] used to say, ‘My body feels like it’s sluggish somehow’ and then he has the chum dry fish and he said it revives him because of the nutrients from that fish” (ANV10). Another Elder acknowledged the long-term benefits and said “Their [ancestors’] longevity was a result of eating their own traditional food that kept them healthy” (ANV7).

To further illustrate the superiority of traditional foods, Elders compared the value of traditional foods to those bought at the store. One Elder stated that “All this food, wild food, is organic, better than store bought food” (ANV4). Another Elder said, “The most important Yup’ik food that we eat today, they have a lot of good stuff in them, vitamins, healthier food. They’re more healthier than the ones we eat like when we go into Bethel [a larger community with restaurants and stores], like junk food,” (ANV10).

Given the benefits of traditional foods, the Elders also expressed the importance of introducing traditional foods such as meat broth, fish, berries, and greens to young children, starting during pregnancy. As one shared, “The mom, when they are pregnant, they need to start right there in eating traditional foods and stay away from processed foods or junk food when they are pregnant. I think that’s where it starts, when they smell that food” (ANV2). This is important because they believe, “Native food is healthy, it’s good for a child” (ANV5) and that, “When you start, when you have our foods right from the beginning, it affects your brain, your growth, your whole body, the way you act” (ANV4). Many connected these early experiences with preferences later on. “He’ll eat all kinds of Native food because we started him young” (ANV1).

*Like I said, in the beginning, what we eat from when you are little, like even infants, if you want to train them to eat blackfish or Native foods and stuff, they take the food and put it into their mouth. As they are growing, that is what they are going to eat. They will be able to eat it because they recognize the taste. That is what they want* (ANV4).

A number of Elders shared how they instilled a preference for traditional foods, which also helped them identify potential allergies early on. Some commented about wiping food on the baby’s mouth to help them, “start to eat Eskimo food” (ANV5) or help them smell what they are cooking so they know what it is “before they even start growing” (ANV2). One further explained,

*Even when they’re newborns, we kind of let them taste from the mouth. Like my oldest daughter, when she was a newborn, I let her taste some, it was very bloody, my fingers were very bloody from seal liver. As she was growing, I was surprised she loved seal liver. She liked to eat it with seal oil, raw liver. We eat it because I put it on her lips. To me, it’s up to the parent to teach that five-year old about our Native food* (ANV5).

### Cultural benefits of traditional foods

Elders also expressed the importance of eating traditional foods to retain and promote a cultural identity. For example, families celebrated “first catch parties”, where a child’s first collection of berries or their first fish or animal caught (moose, bird, duck), were given to Elders (ANV6) or was celebrated in a large community feast (ANV9). One expressed that, “You are proud, and happy, and nice because you know that you are going to do something for your family when you are gathering food” (ANV4). As a result, as one Elder remarked, “Eat your Yup’ik food, don’t lose it” (ANV7).

The connection between traditional foods and culture was shared through stories of learning how to harvest those foods themselves, learning what is safe to eat, and how to not overharvest. It was common to hear an Elder share statements like, “As I was growing up I used to watch my mom prepare food. I learned from watching and touching” (ANV9). This sharing of traditional knowledge is connected to preserving one’s culture, as one Elder stated,

*I think it’s very important that you teach the young people our traditional way of life. That way, they will use it when they get older and pass it onto their children. My father passed on his tradition to me and I’m slowly passing that tradition onto my children. It’s very important* (ANV1).

Traditional knowledge was also tied to pragmatic and essential information needed for sustainability. During a discussion around some of the specific practices that needed to be taught regarding the importance of not over-harvesting, one Elder shared,

*You cannot take all of them (mouse food). They always tell us, you have to save some for them (the mice) because they already provide you with it. You go to another one [den] and then take some more. So there is a tradition too, they have to know that traditional knowledge at the same time to gather those foods* (ANV4).

Additionally, Elders expressed an interest in sharing their knowledge around subsistence practices like how to find, prepare, store, and cook traditional foods, which includes showing specialized ways of taking care of fish, harvesting berries and greens, braiding grass, preparing nets, and setting traps for, “If you don’t teach them how to catch their food, they won’t know it” (ANV7).

### Concerns about changing dietary patterns

The tenor of the conversations amongst the Elders often turned when the focus changed from what was done in the past to present time, as many expressed concerns about the current generation of parents and children. In particular, they shared angst over their lack of knowledge and consumption of traditional foods, their approaches to parenting, the perceived lack of physical activity, and their inability to pass on the traditional ways of knowing. Following are the sub-themes that emerged in the conversations.

#### Traditional Foods

Elders’ enthusiastic recollections of how they collected and shared traditional foods as children turned solemn as they openly expressed concern that the current generation of parents may lack the knowledge to keep their children healthy. “Nowadays, the younger generation eats nothing but junk foods” (ANV5) and as a result, “These young ones that don’t know, hardly eat any Native food. They just want store-bought meals from the store” (ANV1). This assertion was based on what many seemed to witness, as one mentioned, “When I see the children, I always see them eating chips and candy bars and pop and juice, even though we tell them not to have them all the time, to have Yup’ik food, but they don’t like to eat it” (ANV6). Another reiterated that experience by saying, “My concern is when I observe the store, they stock up a lot of soft drinks like pop, sugary stuff, and they disappear in no time … So that kind of tells us they’re eating a lot of that stuff” (ANV10).

Elders expressed concern that the shift in dietary patterns will negatively affect the health of their families. “The results of eating so much sweet/sugary food are not good. It is common to see people with pop in their hand and drinking it. The results are detrimental...Drinking that soda does not have a good end” (ANV7). And, this issue was one they saw as being an imminent concern.

*I think today a lot of our kids are consuming too much sugar. That’s really bad for them and causes a lot of problems for your health. And then I’ve never seen so much disease, white man disease compared to 50 years ago. Today cancer is probably the number one killer. I’m kind of afraid that diabetes is probably going to come up because a lot of these kids are consuming too much sugar, a lot of potato chips, and other stuff.* (ANV9).

This stood in stark contrast to the ways the Elders grew up.

*Growing up in spring camps, summer camps, and here, we depended on fish just like they said, moose and everything that is provided for us here. And I think that is why there was hardly any deaths here. People were very healthy and strong and lack of dental problems in the future and lack of, no diabetes problems because of the health foods. I think the changes with the federal government coming and starting to feed us processed foods and others really changed our life. And then the food stamps came and then people started buying processed foods or getting the processed foods and that in a very short period of time, it went from very healthy people into you know, getting dependent on somebody else’s rules of nutrition. When BIAs [Bureau of Indian Affairs Schools] came over and started the USDA [United States Department of Agriculture] and we just started to eat food in the schools and it was different from our diet at our home. It really changed* (ANV2).

But, many also seemed to believe that part of the issue was the current generation’s lack of understanding of why traditional foods were different from those they got at the store.

*I think one of them [child] was 3 years old, they asked me how come I never buy them chips. ‘That’s not real food, it’s just junk food’ is what I answered. And I told them, ‘The food I cook that Daddy catches from the land and ocean and that I pick with you kids has a lot of vitamins and iron* (ANV5).

Elders also acknowledged that many new parents did not know how to harvest and gather these traditional foods but instead, got most of their food from the store (ANV6) or relied on food stamps (ANV11). “Many of our young people don’t know how to go out there and catch something, fish. They don’t know the land, too” (ANV1). They worried about this turning into a long-term problem because, “Eating Yup’ik food, it’s up to the parents really. They need to encourage their kids to eat healthier foods” (ANV10).

#### Drivers of changing dietary patterns

Some of Elders’ reflections seemed to be related to forces beyond their control. For example, several Elders worried that traditional early schooling does not reinforce Native ways. “These kids are going to have Head Start too early. They should have been home longer and they should start school at a later date and should be taught at home” (ANV1). They found it problematic that the schools and USDA have different standards of acceptability when it comes to donated foods than families use for themselves, which is compounded by the fact that there are limited commercial facilities for traditional food processing. Elders also stated that the negative impacts from missionaries and teachers were still present and felt in their traditional foods and ways. As one elaborated,

*But one of the sad things that occurred was especially before the ‘50s, the 1950s, the old people were still, the teachers were young people at home and in the community. But at some time, we were told, the young people were told to quit speaking Yup’ik. And our Elders didn’t speak English, only Yup’ik. So as a result, they more or less started quieting down and not teaching what they know. So again, people didn’t get the same teachings about living, about values, about gathering that the Elders received. And maybe that’s why many of our young people don’t know how to live in the right way* (ANV1).

Elders also believed that western schools, like Head Start, which uses a family-style approach that allows children to serve themselves, teaches poor habits that contribute to food waste.

*I think one of the things that is not too good for our young people is the way the schools serve food. They waste a lot of food. Kids are too … They eat a little bit of this and throw the rest away. But not like he said, we eat what we’re given and we eat it respectfully* (ANV1).

Elders spoke of influences that today’s generations have to contend with that the Elders did not, such as sugar sweetened beverages, which are more convenient, and less expensive than healthier alternatives. Many children are introduced to sugar sweetened beverages including powdered drinks (e.g. Tang) and soda at an early age through bottles and sippy cups. Elders indicated they [parents] used the sugar sweetened beverages as a reward, as a babysitter, or to keep children quiet. “Like they start crying and they stick a lollipop in their mouth and sit them in front of the TV and that’s it. They’re quiet. And leave them and go Bingo” (ANV11).

Another outside, western influence they identified was the use of screens, like televisions, smart phones, iPads, and video games. “One thing I learned is the parents who have the television babysit their kids, they’re English speakers. They grow up as English speakers from watching TV so much, probably from when they were in their walker” (ANV12). While this Elder connected outside, western influence to the use of English over Yup’ik language, it was also connected to concerns around physical activity, “We had a lot of activities when we were younger in those days because without technologies [of] today, we have fast pace” (ANV1). Another Elder also suggested it seemed to replace more traditional forms of activity, to the detriment of children’s health.

*From all the way to eighth grade, we had all these lap games, outdoors, indoor games, all these different activities that these guys had to grow up with. We didn’t understand that those games were to keep us physically fit and healthy. I see the change. My generation, none of our kids, my own kids, I had to teach them how to play these games and teaching these kids the games and explaining that the younger they are, the healthier they will be in high school. But looking at kids nowadays, my age group, we were healthy, fit, not obese. We had muscles. We were working out. We breathed okay. We were physically fit all the way through high school. This generation you see obesity. Kids are very sickly. It’s just totally the opposite. They’re not physically fit. They go up the stairs and they’re huffing and puffing. Me and some parents are like, ‘Gee, when I was their age, I was able to do these things, and my own child doesn’t do these things’”* (ANV5).

This stood in stark contrast to how they were raised, as the Elders didn’t worry about not being active because when they were children, they spent much of their time doing chores and playing games.

### Suggestions to maintain subsistence practices

Elders articulated a desire to help new parents learn how to live better, raise healthier children, and find balance in life.

*I’d sure like to see these kids being taught from Elders about how they used to live, the Native [way], especially the language and all the stuff they did about hunting or helping people that cannot do things, like Elders or people with disabilities* (ANV9).

Elders mentioned a number of sources of knowledge that could be utilized to promote traditional foods, including a university that has documented the nutritional value of their traditional food, schools and churches that provide cultural camps, and organizations like Head Start that can be used to talk about, serve, and reinforce traditional foods. As one stated, “I think if you start talking to the Head Start students at a very young age in how to respect another person’s property, then another person, I think the village in the long run would be a lot better” (ANV1). In sum, the Elders wanted to work with the new generation of young parents to ensure their Native ways of knowing survived, even if that knowledge did not come straight from them.

## Discussion

Elders expressed perspectives related to maintaining a healthy and traditional lifestyle. They consistently espoused the importance of eating traditional foods, such as seal oil, fish, berries, and tundra greens, connecting these foods with both good nutrition and the practices and values of subsistence cultures. They expressed a number of concerns about changing values and practices around subsistence and whether the current generation possessed the ability to harvest and eat traditional foods. Elders voiced concern about young people who appear to rely on processed, western foods and their ability to raise their children with values consistent with their ancestors. They described a number of external influences that may precipitate changing dietary patterns and negatively impact of the health of children, including government assistance programs; ready access to sugar sweetened beverages and junk food; and an abundant use of screen technologies (e.g., televisions, iPads, and phones). Despite these concerns, Elders recognized opportunities for sharing their historical knowledge and traditional wisdom, and a desire to be a positive force during this time of great change.

During the past 60 to 70 years, AN people have experienced cultural transition characterized by the integration of western values and culture into their traditional lifestyle [27–29]. Historical events experienced by AN people include loss of land and land-based resources; decimation of the population through epidemics of influenza and tuberculosis; and disruption of families when children were sent to boarding schools for formal education. These events have led to losses in transmission of language and culture [10, 17, 30]. In particular, Elders expressed concern that customs and practices surrounding diet that previously formed the backbone of AN culture, such as hunting, fishing, harvesting, gathering, preserving and sharing food, are disappearing.

Elders insisted children needed to be exposed to traditional AN foods at a very young age so they develop a taste for them. Nutrition science enforces these Elders’ observations, reporting that young children are especially vulnerable to parental food choices, becoming accustomed to foods they are introduced to early in life [31]. Food preferences are established prior to age 6 years and eating habits developed by this age affect lifelong eating behaviors. Currently, few AN children, youth, or young adults consume traditional foods as the basis of their diet and, as a result, have experienced a decrease in diet quality as compared to Elders [32–36].

Elders also espoused their belief that traditional foods coming directly from the local surroundings are healthier than store bought foods. Again, research supports this belief. A growing body of evidence has shown that consuming traditional foods have higher nutrient levels than store bought foods [23, 27–29, 32, 37–40]. In a study by Bersamin et al. of Yup’ik AN people living in the Y-K region, traditional foods accounted for 22% of total energy intake, and participants consuming the most traditional foods had higher nutrient levels [23]. A similar study by Sharma et al. [39] investigated the dietary quality the meals of Yup’ik women living in western Alaska and found that while store bought foods were the most frequently reported food items consumed and sugar-sweetened beverages were the main contributors to energy, traditional foods contributed substantially to protein, iron and vitamin A intakes.

Elders identified a major shift in the foods they received as children and the foods and beverages parents and caregivers provide children today, which the literature supports as well. The modern AN diet is now primarily comprised of store bought, highly processed foods containing large percentages of carbohydrates and saturated fats, highly palatable and manufactured for maximum taste preferences [38]. As AN Elders report, AN children and families consuming these non-traditional foods are experiencing adaptations in taste preferences away from traditional foods. The introduction of manufactured western foods has resulted in different food preferences across generations; Elders consume more traditional foods and fewer processed foods and SSBs than younger adults, youth, and children [28, 36]. Through their own personal eating habits, young AN parents perpetuate the diverging taste preferences of the next generation by introducing processed foods during infancy, which is rightfully a concern, as it is an important driver of increased weight in early childhood [41].

### Limitations

Results of this study should be discussed in light of the following potential limitations. First, while the analysis of the data followed a rigorous process to ensure the results were reliable, member-checking was not carried out with the participants because no identifying or contact information was collected. Ideally, the Elders would have reviewed all findings to ensure their contributions were interpreted correctly; however, the regional review board, comprised of Alaska Native peoples from the region, did review and approve this manuscript per Tribal policy. Finally, while it cannot be assumed that the perspectives shared represent the views of all Yup’ik, Indigenous, or Alaska Native peoples, these perspectives do reflect the views of Elders in the communities in which this project is being developed and implemented.

## Conclusions

The risk of obesity in Alaska Native children is high and prevention efforts should be grounded in local knowledge and values. Yup’ik and Cup’ik Elders in the Y-K Delta area of Alaska place a strong value on practicing a subsistence lifestyle as a source of good nutrition and a critical link to cultural identify. The Elders also voiced concern about external influences that negatively impact the health of children, including government assistance programs; ready access to sugar sweetened beverages and junk food; and an abundant use of screen technologies. Although they do not represent all Alaska Native peoples, their views provide a better understanding on local views of the importance of maintaining a traditional lifestyle.

## Data Availability

Data collected and analyzed for this study are the property of Alaska Native people and held in trust by the Alaska Native Tribal Health Consortium and the University of Alaska Fairbanks. Data are available only upon approval by the Alaska Native Tribal Health Consortium and the Yukon-Kuskokwim Health Corporation.

## Abbreviations

AN: Alaska Native
AN/AI: Alaska Native/American Indian
ANV: Alaska Native Village
BIA: Bureau of Indian Affairs
USDA: United States Department of Agriculture
Y-K: Yukon-Kuskokwim
